# Liver fibrosis assessed via noninvasive liver fibrosis scores is associated with atherosclerotic cardiovascular disease in middle-aged and older patients with prediabetes

**DOI:** 10.3389/fendo.2025.1712481

**Published:** 2026-01-09

**Authors:** Ying Huang, Yuan Yuan, Huixian Wu, Zhiyuan Zhou, Lianhong Li, Xiaolong Huang, Letong Xin, Jingru Li, Junhan Zhang, Caiping Liu, Qun Zhu

**Affiliations:** 1 Department of Endocrinology, The Second Affiliated Hospital of Nanjing Medical University, Nanjing, Jiangsu, China; 2 Health Management Center, The Second Affiliated Hospital of Nanjing Medical University, Nanjing, Jiangsu, China; 3 Guangxi Key Laboratory of Molecular Medicine in Liver Injury and Repair, The First Affiliated Hospital of Guilin Medical University, Guilin, Guangxi, China; 4 Hospital Acquired Infection Control, The Second Affiliated Hospital of Nanjing Medical University, Nanjing, Jiangsu, China

**Keywords:** atherosclerotic cardiovascular disease, liver fibrosis score, liver fibrosis, metabolic dysfunction-associated steatotic liver disease, prediabetes

## Abstract

**Background:**

Metabolic dysfunction-associated steatotic liver disease-related liver fibrosis is linked to an increased risk of atherosclerotic cardiovascular disease (ASCVD); however, its impact on patients with prediabetes remains unclear. The aim of our study was to investigate the associations between ASCVD and three noninvasive liver fibrosis scores, including the fibrosis-4 index (FIB-4), nonalcoholic fatty liver disease fibrosis score (NFS), and aspartate aminotransferase-to-platelet ratio index (APRI), in middle-aged and older patients with prediabetes.

**Methods:**

In this cross-sectional study, a total of 7,409 patients with prediabetes aged 40 to 80 years were classified into two groups based on their ASCVD statuses. Logistic regression analysis was performed to explore the relationships between liver fibrosis scores and ASCVD. Restricted cubic spline (RCS) curves were employed to explore potential nonlinear relationships. The performances of the liver fibrosis scores were compared using receiver operating characteristic (ROC) curves.

**Results:**

Noninvasive liver fibrosis scores, including FIB-4, NFS, and APRI values, were independently associated with an increased likelihood of having ASCVD in middle-aged and older patients with prediabetes. After adjusting for confounding factors, the patients in the high-risk FIB-4, NFS, and APRI groups had 1.5, 2.6, and 1.5 times the odds of ASCVD, respectively, compared with those in the low-risk groups, with odds ratios and 95% confidence intervals of 1.497 (1.089–2.055) for FIB-4 (P = 0.013), 2.632 (1.949–3.560) for NFS (P < 0.001), and 1.483 (1.092–2.002) for APRI (P = 0.011). RCS analysis revealed a nonlinear positive correlation between FIB-4 and ASCVD, whereas the relationships between NFS and ASCVD and between APRI and ASCVD were approximately linear. In the ROC curve analysis, both FIB-4 and NFS exhibited area under the curve values exceeding 0.7 in detecting individuals with ASCVD, whereas APRI was a relatively poor discriminator of ASCVD.

**Conclusions:**

Liver fibrosis assessed via noninvasive indices was significantly positively associated with an increased risk of ASCVD in middle-aged and older patients with prediabetes, thereby underscoring the value of liver fibrosis scores as reliable indicators for identifying the presence of ASCVD in individuals with prediabetes.

## 1 Introduction

Prediabetes refers to an intermediate metabolic state between normoglycemia and diabetes. According to the American Diabetes Association (ADA) criteria, the prevalence of prediabetes is 35.2% among the general adult population in China; moreover, in middle-aged and older populations, the prevalence is nearly 50% (1). Approximately 5–10% of individuals with prediabetes progress to diabetes on an annual basis; over time, up to 70% of those with prediabetes will eventually develop diabetes (2). Atherosclerotic cardiovascular disease (ASCVD) is the leading cause of morbidity and mortality worldwide, imposing a considerable burden on public health. Emerging evidence indicates that, in addition to the well-established association between diabetes and elevated ASCVD risk, prediabetes is also recognized as a significant risk factor for ASCVD, contributing to increased risks of atherosclerosis, ASCVD, and ASCVD mortality (3–5).

Metabolic dysfunction-associated steatotic liver disease (MASLD), formerly known as nonalcoholic fatty liver disease, has emerged as the most common chronic liver disease worldwide and comprises a spectrum of histological types, with liver fibrosis representing one of the most severe types and being the main determinant of disease progression (6). Currently, liver biopsy remains the gold standard for the diagnosis and staging of liver fibrosis; however, its invasiveness limits its use in routine clinical practice. Therefore, noninvasive liver fibrosis scores, such as the fibrosis-4 index (FIB-4), nonalcoholic fatty liver disease fibrosis score (NFS), and aspartate aminotransferase-to-platelet ratio index (APRI), are widely used as surrogates for the screening and risk stratification of individuals who are at high risk of liver fibrosis (6).

Numerous studies have demonstrated that MASLD and the associated development of liver fibrosis are related to an increased risk of ASCVD, and the ability of liver fibrosis scores to identify patients with ASCVD has been evaluated in the general population, as well as in patients with MASLD and those with type 2 diabetes mellitus (T2DM) (7–9). However, the associations between liver fibrosis scores and ASCVD have not been investigated in patients with prediabetes, particularly in middle-aged and older individuals who are at elevated risks for both prediabetes and ASCVD. Hence, we conducted this study to determine the associations between three liver fibrosis scores (FIB-4, NFS, and APRI) and ASCVD in patients with prediabetes aged 40 to 80 years, with an aim of providing valuable insights into the early diagnosis and intervention of this disease.

## 2 Methods

### 2.1 Study population

**Figure 1:**
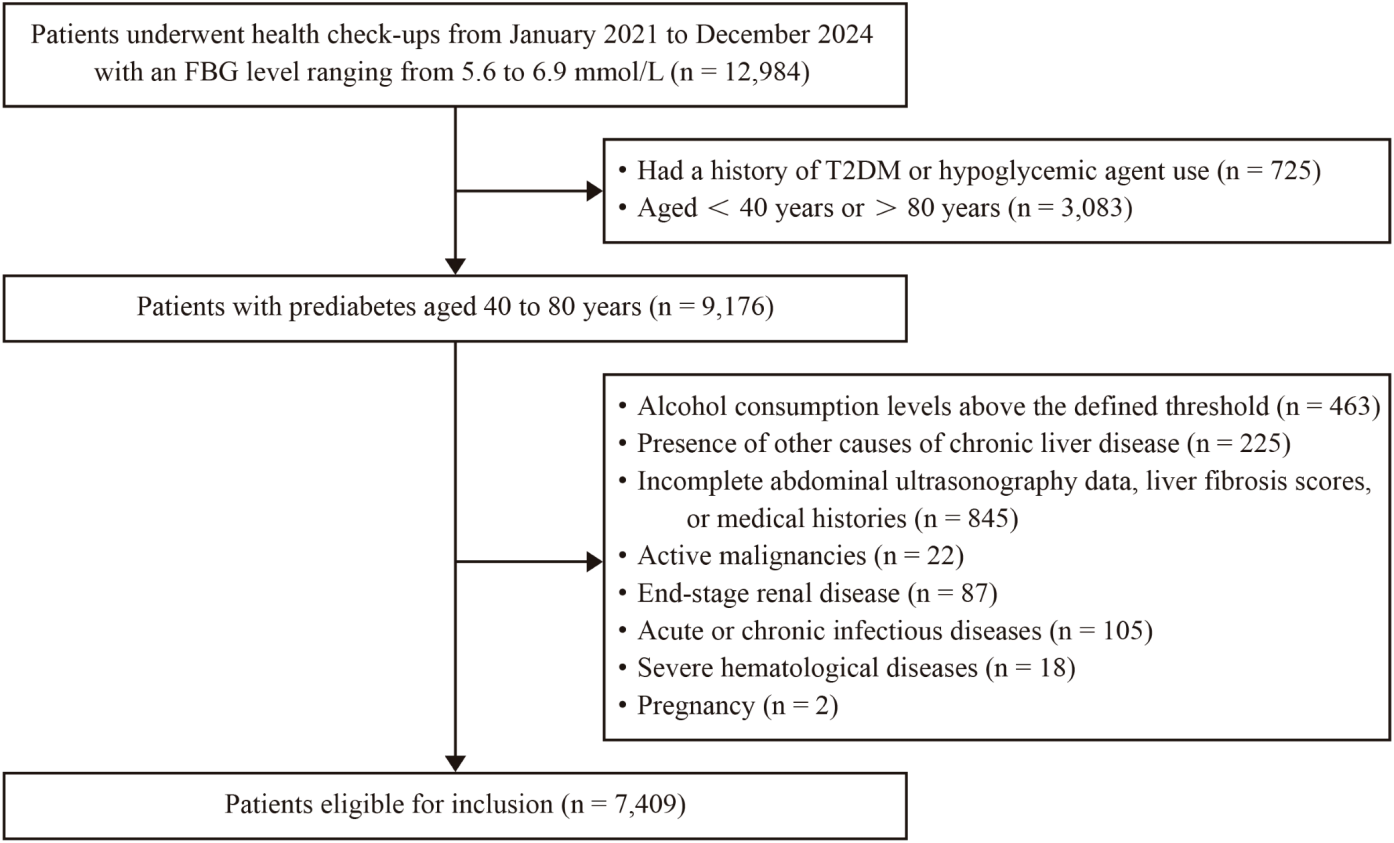
Flowchart of the study participants.

As illustrated in the presented flowchart (**Figure 1**), in our study, we initially screened patients with prediabetes aged 40 to 80 years, all of whom underwent health check-ups at the Second Affiliated Hospital of Nanjing Medical University from January 2021 to December 2024. Patients with alcohol consumption levels exceeding 30 g/day for men and 20 g/day for women, as well as other causes of chronic liver diseases (such as viral hepatitis, autoimmune liver diseases, chronic use of potentially hepatotoxic drugs and liver cirrhosis), were excluded from the study. Other exclusion criteria included incomplete abdominal ultrasonography data, liver fibrosis scores, or medical histories, as well as active malignancies, end-stage renal disease, acute or chronic infectious diseases, severe hematological diseases, and pregnancy. Ultimately, a total of 7,409 participants were enrolled in the study. This study was approved by the Ethics Committee of the Second Affiliated Hospital of Nanjing Medical University (2024-KY-207-01). All of the participants provided written informed consent.

### 2.2 Clinical and laboratory data

The demographic and clinical characteristics of the patients, including their ages, sexes, personal and family histories of disease, medication use, smoking statuses, alcohol consumption levels, and regular exercise behaviors, were collected and documented by professionally trained personnel. Anthropometric measurements were performed by trained physicians following the PhenX Toolkit (10). Height and weight were measured using a calibrated OMRON HNH-219 device, with participants wearing light clothing and standing barefoot. Measurements were taken in duplicate for all participants, and a third measurement was performed if the first two differed beyond predefined thresholds; the average of the two closest measurements was used for analysis. The body mass index (BMI) of each patient was calculated via the following formula: BMI = weight (kg)/height² (m²). Blood pressure (BP) was measured using a validated automatic device (OMRON HBP-9020) in accordance with the 2017 American Heart Association guidelines on high blood pressure in adults (11). Participants rested quietly for 5–10 minutes before measurement. At least two readings of systolic (SBP) and diastolic blood pressure (DBP) were obtained at intervals of 1–2 minutes, and the mean value was used for analysis.

Laboratory measurements were performed on venous blood collected in the morning after an overnight fast of at least 8 hours. Platelet counts (PLT) were measured using EDTA-anticoagulated whole blood on a SYSMEX XN-350 automated hematology analyzer. Biochemical parameters, including fasting blood glucose (FBG), total cholesterol (TC), triglycerides (TG), high−density lipoprotein cholesterol (HDL-C), low−density lipoprotein cholesterol (LDL-C), alanine aminotransferase (ALT), aspartate aminotransferase (AST), albumin, gamma-glutamyl transpeptidase (GGT), blood urea nitrogen (BUN), creatinine, and uric acid, were measured on serum samples obtained by centrifugation using a Roche cobas 8000 automated biochemistry analyzer. All laboratory measurements were performed with internal quality control and external quality assessment, and reference ranges were applied according to the laboratory’s validated procedures, all in accordance with ISO 15189 standards (12). The AST/ALT ratio was calculated, and the estimated glomerular filtration rate (eGFR) was determined using the CKD-EPI equation.

### 2.3 Assessment of hepatic steatosis and advanced fibrosis

MASLD was defined as the presence of hepatic steatosis, as confirmed via imaging, with no evidence of any other discernible secondary cause and accompanied by at least one cardiometabolic risk factor (such as excess weight, obesity, or waist circumference > 95th percentile; hypertension; prediabetes or T2DM; elevated TG; or low HDL-C) (13). The diagnosis of MASLD also required either no alcohol consumption or alcohol intake levels below hepatotoxic thresholds (< 30 g/day for men and < 20 g/day for women). The presence of hepatic steatosis was determined based on abdominal ultrasonography (PHILIPS Affiniti 50 system) performed by experienced radiologists, who were blinded to the participants’ details. Due to the fact that all of the participants in our study involved patients with prediabetes, any individual identified as having hepatic steatosis was classified as having MASLD.

We used the following three liver fibrosis scores to assess the degrees of advanced fibrosis suffered by the patients: (1) FIB-4 = (age × AST)/(PLT × ALT^1/2^) (age in years, ALT and AST in U/L, and PLT in 10^9^/L); (2) NFS = -1.675 + 0.037 × age + 0.094 × BMI + 1.13 × impaired fasting glucose or diabetes (yes = 1, no = 0) + 0.99 × AST/ALT - 0.013 × PLT - 0.66 × albumin (age in years, BMI in kg/m^2^, AST and ALT in U/L, PLT in 10^9^/L, and albumin in g/dL); and (3) APRI = (AST/ULN × 100)/PLT (AST in U/L and PLT in 10^9^/L; ULN was defined as the upper limit of the normal range for AST, with ULN = 40 U/L in our study). Advanced fibrosis risk was categorized according to established cutoffs for each index: low, indeterminate, and high risk were defined as < 1.3, 1.3–2.67, and ≥ 2.67 for FIB-4; < -1.455, -1.455–0.676, and ≥ 0.676 for NFS; and < 0.25, 0.25–0.5, and ≥ 0.5 for APRI, respectively (14).

### 2.4 Definitions

Prediabetes was defined as an FBG level ranging from 5.6 to 6.9 mmol/L among individuals without a history of T2DM or hypoglycemic agent use, in accordance with the guidelines of the ADA (15). ASCVD was defined as the presence of coronary heart disease (CHD), ischemic stroke, or both. The CHD diagnoses were based on self-reported medical histories verified through a review of the supporting medical records, including prior myocardial infarction, evidence of coronary stenosis > 50%, angina pectoris, and prior coronary revascularization procedures. The ischemic stroke diagnosis process was based on self-reported medical histories confirmed by medical records or brain imaging data, including computed tomography and magnetic resonance imaging scans. Hypertension was defined as self-reported antihypertensive agent use and/or a measured BP ≥ 140/90 mmHg. Individuals were classified as smokers if they self-reported having smoked ≥ 100 cigarettes in their lifetime, regardless of their current or former smoking statuses. We defined mild alcohol consumption as drinking less than three times per week. Regular exercise was defined as high-intensity physical activity (causing extreme shortness of breath) performed for at least 20 minutes at a frequency of three times per week or moderate-intensity physical activity (causing substantial shortness of breath) performed for at least 30 minutes at a frequency of five times per week.

### 2.5 Statistical analysis

Categorical variables are presented as numbers with percentages, and continuous variables are presented as either means ± standard deviations or medians with interquartile ranges (depending on their distributions). The differences between categorical variables were assessed using the chi-square test, and the differences between continuous variables were assessed using either Student’s t test for normally distributed variables or the Mann-Whitney U test for nonnormally distributed variables. Univariate and adjusted logistic regression models were developed using the enter method, with odds ratios (ORs) and 95% confidence intervals (CIs) calculated. The liver fibrosis scores were analyzed as continuous variables and categorical variables with conventional cutoff values (as described above). Additionally, we employed a restricted cubic spline (RCS) analysis with three knots to explore potential nonlinear relationships. For subgroup analysis purposes, we stratified the data by age, BMI score, hypertension status, smoking status, and family history of ASCVD, and the interactions between the subgroups were assessed. Receiver operating characteristic (ROC) curves were used to assess the accuracies of the three liver fibrosis scores in terms of identifying ASCVD, CHD, ischemic stroke, and combined CHD and stroke. The area under the curve (AUC) was utilized, and the sensitivity, specificity, and cutoff values at the maximal Youden index were calculated to compare the performances of the liver fibrosis scores. Furthermore, we conducted a sensitivity analysis to confirm the reliability of the associations. Sensitivity test I excluded the extreme values of the liver fibrosis scores, which were defined as FIB-4 and/or NFS and/or APRI values below and above the 1st and 99th percentiles, respectively. Sensitivity test II excluded patients with elevated levels of ALT and/or AST, which were defined as ALT ≥ 40 U/L and/or AST ≥ 40 U/L. All of the reported P values were two-sided, and P < 0.05 was considered to be statistically significant. Statistical analyses were performed using R version 4.4.2 and IBM SPSS Statistics 25.

## 3 Results

### 3.1 Clinical and laboratory characteristics of the study population

This study included 7,409 patients with prediabetes, of whom 49.8% were male, with a mean age of 58.1 years. A total of 1,376 (18.6%) patients had previous histories of ASCVD, including 741 (10.0%) with CHD, 396 (5.3%) with ischemic stroke, and 239 (3.2%) with combined CHD and stroke. **Table 1** provides a summary of the clinical and laboratory characteristics of the patients based on their ASCVD statuses. The patients in the ASCVD group were older than those without ASCVD and were more likely to have hypertension, use antihypertensive and lipid-lowering treatments, smoke, and have family histories of ASCVD; however, they were less likely to participate in regular exercise (all P < 0.05). The group with ASCVD also exhibited higher BMI, SBP, DBP, FBG, AST, AST/ALT ratio, BUN, and uric acid levels, as well as lower TC, TG, HDL-C, LDL-C, PLT, albumin, and eGFR levels (all P < 0.05). No statistically significant differences were observed for sex, mild alcohol consumption, or ALT or GGT levels (all P > 0.05). Furthermore, a total of 4,018 individuals in our study were identified as having hepatic steatosis, accounting for 54.2% of the study population. Despite the overall high prevalence of this condition, no difference was observed between the proportions of hepatic steatosis in the two groups. In contrast, the patients in the ASCVD group exhibited higher liver fibrosis scores (FIB-4, NFS, and APRI values), and the differences were observed to be highly statistically significant (all P < 0.001).

**Table 1:** Clinical and laboratory characteristics of the entire study population (n = 7409).

Variables	Patients without ASCVD (n = 6033)	Patients with ASCVD (n = 1376)	P value^a^	Patients with CHD (n = 741)	P value^b^	Patients with ischemic stroke (n = 396)	P value^c^	Patients with combined CHD and stroke (n = 239)	P value^d^
Age, years	56.04 ± 10.09	67.26 ± 8.47	<0.001	65.91 ± 9.16	<0.001	68.16 ± 7.29	<0.001	69.96 ± 7.15	<0.001
Male, n (%)	2994 (49.6)	699 (50.8)	0.433	379 (51.1)	0.435	197 (49.7)	0.963	123 (51.5)	0.577
BMI, kg/m^2^	24.68 ± 3.26	25.20 ± 3.26	<0.001	25.32 ± 3.25	<0.001	25.02 ± 3.27	0.041	25.13 ± 3.30	0.033
SBP, mmHg	132.68 ± 17.35	140.57 ± 18.24	<0.001	139.95 ± 17.85	<0.001	141.54 ± 18.82	<0.001	140.92 ± 18.46	<0.001
DBP, mmHg	80.92 ± 11.10	82.05 ± 11.52	<0.001	81.62 ± 11.39	0.110	82.35 ± 11.64	0.013	82.90 ± 11.75	0.007
FBG, mmol/L	5.90 (5.70, 6.10)	5.90 (5.70, 6.20)	<0.001	5.90 (5.70, 6.20)	0.003	5.90 (5.70, 6.15)	0.169	5.90 (5.70, 6.20)	0.007
TC, mmol/L	5.11 ± 0.96	4.61 ± 1.14	<0.001	4.79 ± 1.15	<0.001	4.47 ± 1.15	<0.001	4.31 ± 1.04	<0.001
TG, mmol/L	1.43 (1.04, 2.05)	1.35 (1.02, 1.86)	<0.001	1.40 (1.07, 1.95)	0.650	1.27 (0.98, 1.80)	<0.001	1.24 (0.98, 1.72)	<0.001
HDL-C, mmol/L	1.44 ± 0.38	1.36 ± 0.36	<0.001	1.39 ± 0.36	0.002	1.33 ± 0.37	<0.001	1.31 ± 0.32	<0.001
LDL-C, mmol/L	3.20 ± 0.88	2.75 ± 0.99	<0.001	2.89 ± 1.00	<0.001	2.64 ± 0.99	<0.001	2.49 ± 0.91	<0.001
PLT, ×10^9^/L	235.02 ± 55.41	211.15 ± 52.04	<0.001	212.88 ± 50.99	<0.001	210.03 ± 54.82	<0.001	207.65 ± 50.50	<0.001
ALT, U/L	19.10 (14.10, 28.00)	19.00 (14.15, 26.10)	0.139	20.00 (15.00, 27.70)	0.176	18.25 (13.10, 24.70)	0.006	17.20 (13.50, 23.40)	0.006
AST, U/L	20.80 (17.40, 25.10)	21.40 (18.00, 26.00)	<0.001	22.00 (18.30, 26.50)	<0.001	21.00 (17.00, 25.10)	0.726	21.00 (18.00, 25.60)	0.173
AST/ALT ratio	1.06 (0.82, 1.35)	1.13 (0.91, 1.39)	<0.001	1.08 (0.88, 1.35)	0.007	1.14 (0.90, 1.43)	<0.001	1.20 (1.00, 1.41)	<0.001
Albumin, g/L	46.94 ± 2.47	44.79 ± 4.11	<0.001	46.05 ± 3.50	<0.001	43.88 ± 4.41	<0.001	42.41 ± 3.92	<0.001
GGT, U/L	23.00 (16.00, 37.00)	22.00 (16.00, 36.00)	0.410	24.00 (16.00, 38.00)	0.271	21.00 (16.00, 33.00)	0.059	21.00 (15.00, 32.00)	0.089
BUN, mmol/L	5.22 ± 1.21	5.42 ± 1.34	<0.001	5.43 ± 1.30	<0.001	5.41 ± 1.37	0.010	5.43 ± 1.42	0.029
eGFR, mL/min/1.73m^2^	94.08 ± 14.05	86.22 ± 14.30	<0.001	86.79 ± 14.07	<0.001	87.05 ± 14.15	<0.001	83.06 ± 14.86	<0.001
Uric acid, μmol/L	328.67 ± 81.75	334.85 ± 85.77	0.015	345.04 ± 84.56	<0.001	315.83 ± 83.76	0.003	334.75 ± 87.72	0.261
Hypertension, n (%)	3095 (51.3)	1113 (80.9)	<0.001	578 (78.0)	<0.001	330 (83.3)	<0.001	205 (85.8)	<0.001
Antihypertensive treatment, n (%)	2309 (38.3)	858 (62.4)	<0.001	460 (62.1)	<0.001	244 (61.6)	<0.001	154 (64.4)	<0.001
Lipid-lowering treatment, n (%)	836 (13.9)	485 (35.2)	<0.001	212 (28.6)	<0.001	163 (41.2)	<0.001	110 (46.0)	<0.001
Smoking, n (%)	1501 (24.9)	500 (36.3)	<0.001	273 (36.8)	<0.001	138 (34.8)	<0.001	89 (37.2)	<0.001
Mild alcohol consumption, n (%)	1719 (28.5)	426 (31.0)	0.069	217 (29.3)	0.653	131 (33.1)	0.051	78 (32.6)	0.165
Regular exercise, n (%)	2021 (33.5)	361 (26.2)	<0.001	197 (26.6)	<0.001	103 (26.0)	0.002	61 (25.5)	0.010
Family history of ASCVD, n (%)	923 (15.3)	348 (25.3)	<0.001	178 (24.0)	<0.001	104 (26.3)	<0.001	66 (27.6)	<0.001
Hepatic steatosis, n (%)	3266 (54.1)	752 (54.7)	0.729	444 (59.9)	0.003	193 (48.7)	0.037	115 (48.1)	0.067
FIB-4	1.14 (0.85, 1.54)	1.60 (1.25, 2.09)	<0.001	1.52 (1.22, 2.04)	<0.001	1.63 (1.30, 2.08)	<0.001	1.77 (1.35, 2.24)	<0.001
< 1.3	3755 (62.2)	393 (28.6)	<0.001	243 (32.8)	<0.001	99 (25.0)	<0.001	51 (21.3)	<0.001
1.3–2.67	2111 (35.0)	850 (61.8)		429 (57.9)		264 (66.7)		157 (65.7)	
≥ 2.67	167 (2.8)	133 (9.7)		69 (9.3)		33 (8.3)		31 (13.0)	
NFS	-1.19 ± 1.05	-0.20 ± 1.04	<0.001	-0.37 ± 1.03	<0.001	-0.10 ± 1.01	<0.001	0.16 ± 1.00	<0.001
< -1.455	2446 (40.5)	154 (11.2)	<0.001	109 (14.7)	<0.001	35 (8.8)	<0.001	10 (4.2)	<0.001
-1.455–0.676	3380 (56.0)	956 (69.5)		519 (70.0)		274 (69.2)		163 (68.2)	
≥ 0.676	207 (3.4)	266 (19.3)		113 (15.2)		87 (22.0)		66 (27.6)	
APRI	0.23 (0.18, 0.30)	0.26 (0.21, 0.34)	<0.001	0.26 (0.21, 0.34)	<0.001	0.26 (0.20, 0.35)	<0.001	0.27 (0.21, 0.35)	<0.001
< 0.25	3533 (58.6)	615 (44.7)	<0.001	325 (43.9)	<0.001	185 (46.7)	<0.001	105 (43.9)	<0.001
0.25–0.5	2239 (37.1)	663 (48.2)		359 (48.4)		186 (47.0)		118 (49.4)	
≥ 0.5	261 (4.3)	98 (7.1)		57 (7.7)		25 (6.3)		16 (6.7)	

ASCVD, atherosclerotic cardiovascular disease; CHD, coronary heart disease; BMI, body mass index; SBP, systolic blood pressure; DBP, diastolic blood pressure; FBG, fasting blood glucose; TC, total cholesterol; TG, triglycerides; HDL-C, high−density lipoprotein cholesterol; LDL-C, low−density lipoprotein cholesterol; PLT, platelet counts; ALT, alanine aminotransferase; AST, aspartate aminotransferase; GGT, gamma-glutamyl transpeptidase; BUN, blood urea nitrogen; eGFR, estimated glomerular filtration rate; FIB-4, fibrosis-4 index; NFS, nonalcoholic fatty liver disease fibrosis score; APRI, aspartate aminotransferase-to-platelet ratio index.

Categorical variables are presented as n (%), and continuous variables are presented as the means ± SDs or medians (IQRs). ^a^The P values are derived from the comparison between patients without ASCVD and those with ASCVD. ^b^The P values are derived from the comparison between patients without ASCVD and those with CHD. ^c^The P values are derived from the comparison between patients without ASCVD and those with ischemic stroke. ^d^The P values are derived from the comparison between patients without ASCVD and those with combined CHD and stroke. P < 0.05 is considered to be statistically significant.

### 3.2 Associations of hepatic steatosis, AST/ALT ratio, and three liver fibrosis scores with ASCVD and its subtypes

As shown in **Table 2**, a logistic regression analysis was performed to assess the associations of hepatic steatosis, AST/ALT ratio, and three liver fibrosis scores with ASCVD, CHD, ischemic stroke, and combined CHD and stroke in patients with prediabetes. We did not identify any statistically significant correlations between hepatic steatosis and ASCVD, CHD, ischemic stroke, or combined CHD and stroke in the multivariate regression analysis (all P > 0.05). Evident relevance was observed when the AST/ALT ratio was linked to ASCVD, CHD, ischemic stroke, and combined CHD and stroke in the univariate analysis, with ORs and 95% CIs of 1.374 (1.216–1.550) for ASCVD (P < 0.001), 1.213 (1.033–1.416) for CHD (P = 0.016), 1.414 (1.156–1.713) for ischemic stroke (P < 0.001), and 1.685 (1.335–2.100) for combined CHD and stroke (P < 0.001), respectively; however, these results were observed to be statistically nonsignificant in the adjusted model (all P > 0.05). In contrast, our findings revealed that the risk of having ASCVD gradually increased with increasing liver fibrosis scores via both univariate and adjusted logistic regression models. In the continuous analysis, the univariate ORs and 95% CIs were 2.928 (2.675–3.209) for FIB-4, 2.457 (2.305–2.623) for NFS, and 6.472 (4.388–9.562) for APRI (all P < 0.001). Following adjustments made for the confounding factors, including age, BMI (except for the NFS), FBG, hypertension, TC, TG, HDL-C, lipid-lowering treatment, BUN, eGFR, uric acid, smoking, regular exercise, and family history of ASCVD, the positive associations between the liver fibrosis scores and ASCVD remained consistent, and the corresponding ORs and 95% CIs were 1.260 (1.114–1.425) for FIB-4, 1.342 (1.235–1.459) for NFS, and 2.388 (1.459–3.880) for APRI (all P < 0.001). When the three liver fibrosis scores were categorized into low-risk, indeterminate-risk, and high-risk groups, the associations remained significant. After adjusting for the same confounding factors, the patients included in the high-risk FIB-4, NFS, and APRI groups had 1.5, 2.6, and 1.5 times the odds of ASCVD, respectively, compared with those in the low-risk groups, with ORs and 95% CIs of 1.497 (1.089–2.055) for FIB-4 (P = 0.013), 2.632 (1.949–3.560) for NFS (P < 0.001), and 1.483 (1.092–2.002) for APRI (P = 0.011) being observed.

**Table 2:** Associations of hepatic steatosis, AST/ALT ratio, and three liver fibrosis scores with ASCVD, CHD, ischemic stroke, and combined CHD and stroke in patients with prediabetes.

		Univariate model		Adjusted model^a^	
		OR (95% CI)	P value	OR (95% CI)	P value
ASCVD					
Hepatic steatosis		1.021 (0.908–1.149)	0.729	0.952 (0.812–1.117)	0.547
AST/ALT ratio		1.374 (1.216–1.550)	<0.001	1.049 (0.883–1.243)	0.581
FIB-4	Continuous variable	2.928 (2.675–3.209)	<0.001	1.260 (1.114–1.425)	<0.001
	< 1.3	1 (Reference)		1 (Reference)	
	1.3–2.67	3.847 (3.378–4.389)	<0.001	1.328 (1.121–1.574)	0.001
	≥ 2.67	7.609 (5.920–9.771)	<0.001	1.497 (1.089–2.055)	0.013
NFS	Continuous variable	2.457 (2.305–2.623)	<0.001	1.342 (1.235–1.459)	<0.001
	< -1.455	1 (Reference)		1 (Reference)	
	-1.455–0.676	4.492 (3.772–5.385)	<0.001	1.536 (1.253–1.891)	<0.001
	≥ 0.676	20.410 (16.024–26.107)	<0.001	2.632 (1.949–3.560)	<0.001
APRI	Continuous variable	6.472 (4.388–9.562)	<0.001	2.388 (1.459–3.880)	<0.001
	< 0.25	1 (Reference)		1 (Reference)	
	0.25–0.5	1.701 (1.506–1.922)	<0.001	1.197 (1.037–1.381)	0.014
	≥ 0.5	2.157 (1.678–2.754)	<0.001	1.483 (1.092–2.002)	0.011
CHD					
Hepatic steatosis		1.267 (1.085–1.481)	0.003	1.093 (0.899–1.330)	0.372
AST/ALT ratio		1.213 (1.033–1.416)	0.016	0.973 (0.785–1.198)	0.803
FIB-4	Continuous variable	2.492 (2.242–2.773)	<0.001	1.274 (1.104–1.469)	<0.001
	< 1.3	1 (Reference)		1 (Reference)	
	1.3–2.67	3.140 (2.662–3.712)	<0.001	1.283 (1.043–1.579)	0.018
	≥ 2.67	6.385 (4.666–8.665)	<0.001	1.552 (1.062–2.254)	0.022
NFS	Continuous variable	2.080 (1.926–2.248)	<0.001	1.244 (1.125–1.376)	<0.001
	< -1.455	1 (Reference)		1 (Reference)	
	-1.455–0.676	3.446 (2.796–4.283)	<0.001	1.373 (1.081–1.754)	0.010
	≥ 0.676	12.250 (9.089–16.536)	<0.001	2.143 (1.492–3.078)	<0.001
APRI	Continuous variable	6.703 (4.245–10.537)	<0.001	2.755 (1.586–4.717)	<0.001
	< 0.25	1 (Reference)		1 (Reference)	
	0.25–0.5	1.743 (1.487–2.044)	<0.001	1.262 (1.060–1.503)	0.009
	≥ 0.5	2.374 (1.731–3.209)	<0.001	1.567 (1.098–2.207)	0.012
Ischemic stroke					
Hepatic steatosis		0.805 (0.657–0.987)	0.037	0.832 (0.638–1.084)	0.173
AST/ALT ratio		1.414 (1.156–1.713)	<0.001	0.991 (0.746–1.300)	0.950
FIB-4	Continuous variable	2.718 (2.380–3.104)	<0.001	1.158 (0.942–1.418)	0.160
	< 1.3	1 (Reference)		1 (Reference)	
	1.3–2.67	4.743 (3.756–6.037)	<0.001	1.488 (1.114–1.997)	0.008
	≥ 2.67	7.495 (4.849–11.337)	<0.001	1.294 (0.760–2.167)	0.334
NFS	Continuous variable	2.659 (2.396–2.958)	<0.001	1.416 (1.235–1.625)	<0.001
	< -1.455	1 (Reference)		1 (Reference)	
	-1.455–0.676	5.665 (4.028–8.221)	<0.001	1.749 (1.197–2.618)	0.005
	≥ 0.676	29.372 (19.527–45.080)	<0.001	3.254 (1.986–5.401)	<0.001
APRI	Continuous variable	4.017 (2.104–7.390)	<0.001	1.677 (0.687–3.881)	0.242
	< 0.25	1 (Reference)		1 (Reference)	
	0.25–0.5	1.586 (1.285–1.958)	<0.001	1.117 (0.880–1.416)	0.362
	≥ 0.5	1.829 (1.157–2.778)	0.007	1.416 (0.830–2.332)	0.185
Combined CHD and stroke					
Hepatic steatosis		0.786 (0.606–1.018)	0.068	0.778 (0.556–1.087)	0.141
AST/ALT ratio		1.685 (1.335–2.100)	<0.001	1.324 (0.940–1.828)	0.098
FIB-4	Continuous variable	3.103 (2.660–3.622)	<0.001	1.333 (1.050–1.686)	0.017
	< 1.3	1 (Reference)		1 (Reference)	
	1.3–2.67	5.476 (4.005–7.612)	<0.001	1.348 (0.917–2.000)	0.133
	≥ 2.67	13.667 (8.448–21.808)	<0.001	1.700 (0.931–3.072)	0.081
NFS	Continuous variable	3.249 (2.848–3.723)	<0.001	1.696 (1.423–2.028)	<0.001
	< -1.455	1 (Reference)		1 (Reference)	
	-1.455–0.676	11.796 (6.557–23.941)	<0.001	3.212 (1.718–6.695)	<0.001
	≥ 0.676	77.988 (41.352–163.451)	<0.001	6.510 (3.145–14.647)	<0.001
APRI	Continuous variable	5.252 (2.460–10.562)	<0.001	1.680 (0.565–4.610)	0.332
	< 0.25	1 (Reference)		1 (Reference)	
	0.25–0.5	1.773 (1.357–2.321)	<0.001	1.131 (0.835–1.532)	0.425
	≥ 0.5	2.063 (1.159–3.442)	0.009	1.204 (0.616–2.226)	0.569

AST, aspartate aminotransferase; ALT, alanine aminotransferase; ASCVD, atherosclerotic cardiovascular disease; CHD, coronary heart disease; OR, odds ratio; CI, confidence interval; FIB-4, fibrosis-4 index; NFS, nonalcoholic fatty liver disease fibrosis score; APRI, aspartate aminotransferase-to-platelet ratio index.

^a^The model was adjusted for age, BMI (except for the NFS), FBG, hypertension, TC, TG, HDL-C, lipid-lowering treatment, BUN, eGFR, uric acid, smoking, regular exercise, and family history of ASCVD.

We further investigated the associations between the three liver fibrosis scores and an increased likelihood of having CHD. According to both the univariate and multivariate analyses, patients with higher liver fibrosis scores were at elevated risks of having CHD. After making adjustments for multiple variables as described above, each unit increase in the FIB-4, NFS, and APRI was associated with 27.4%, 24.4%, and 175.5% increases, respectively, in CHD risk; moreover, the ORs and 95% CIs were 1.274 (1.104–1.469) for FIB-4, 1.244 (1.125–1.376) for NFS, and 2.755 (1.586–4.717) for APRI (all P < 0.001). These significant associations persisted when the liver fibrosis scores were considered as three different risk groups. According to the adjusted model, the odds of CHD in the patients included in the high-risk FIB-4, NFS, and APRI groups were 1.6, 2.1, and 1.6 times those in the corresponding low-risk groups, with ORs and 95% CIs of 1.552 (1.062–2.254) for FIB-4 (P = 0.022), 2.143 (1.492–3.078) for NFS (P < 0.001), and 1.567 (1.098–2.207) for APRI (P = 0.012) being observed.

Additionally, our study revealed a significant relationship between NFS and an elevated risk of ischemic stroke. In the continuous analysis, the multivariate OR and 95% CI were 1.416 (1.235–1.625) (P < 0.001), thereby indicating that each unit increase in NFS was associated with a 41.6% increase in the risk of ischemic stroke. After stratifying NFS into three different risk levels, the high-risk NFS group exhibited 3.3 times the odds of ischemic stroke compared with the low-risk group, with an OR and 95% CI of 3.254 (1.986–5.401) (P < 0.001). No significant associations were detected between either FIB-4 or APRI and ischemic stroke in this study.

Similar to ischemic stroke, only NFS was significantly associated with an increased risk of combined CHD and stroke. With a continuous variable, the OR and 95% CI were 1.696 (1.423–2.028) in the multivariate analysis (P < 0.001), thus suggesting that each unit increase in NFS was related to a 69.6% increase in the risk of combined CHD and stroke; additionally, with a categorical variable, the high-risk NFS group had 6.5 times the odds of combined CHD and stroke relative to the low-risk group, with an OR and 95% CI of 6.510 (3.145–14.647) (P < 0.001).

### 3.3 Detection of nonlinear relationships

**Figure 2:**
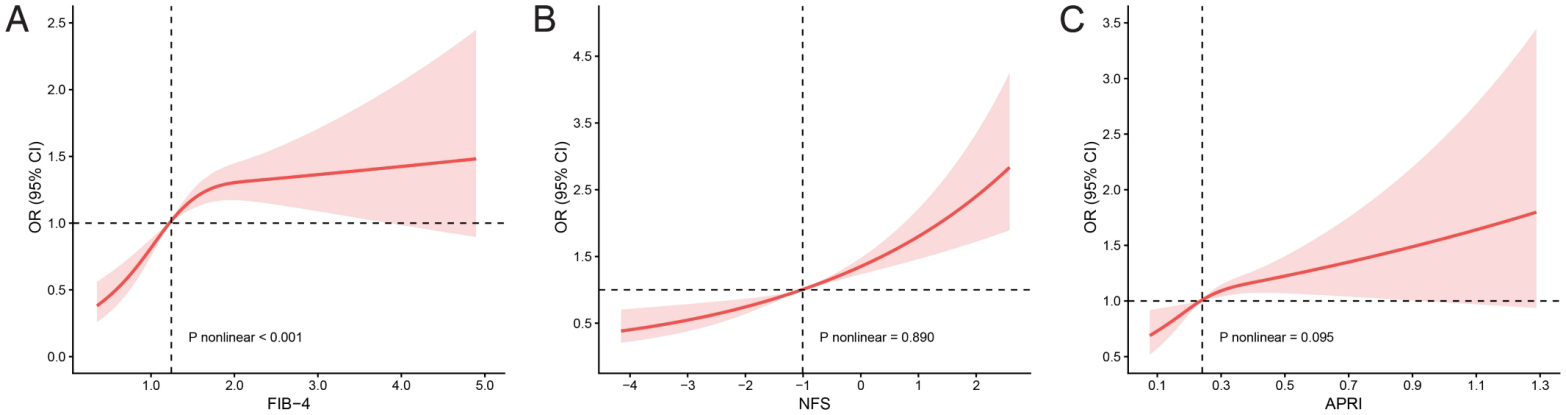
RCS plots of three liver fibrosis scores and the risk of having ASCVD in patients with prediabetes. **(A)** Association between FIB-4 and ASCVD. **(B)** Association between NFS and ASCVD. **(C)** Association between APRI and ASCVD.

We employed RCS curves to assess potential nonlinearity in the relationships between the liver fibrosis scores and ASCVD (**Figure 2**). Our results revealed that there were approximately linear relationships between NFS and ASCVD (P nonlinear = 0.890) and between APRI and ASCVD (P nonlinear = 0.095). In contrast, a nonlinear positive correlation was observed between FIB-4 and ASCVD (P nonlinear < 0.001), with an inflection point at 1.244. Specifically, the ASCVD risk markedly increased with an initial increase in the FIB-4 score, after which it continued to steadily increase.

### 3.4 Subgroup analysis

**Figure 3:**
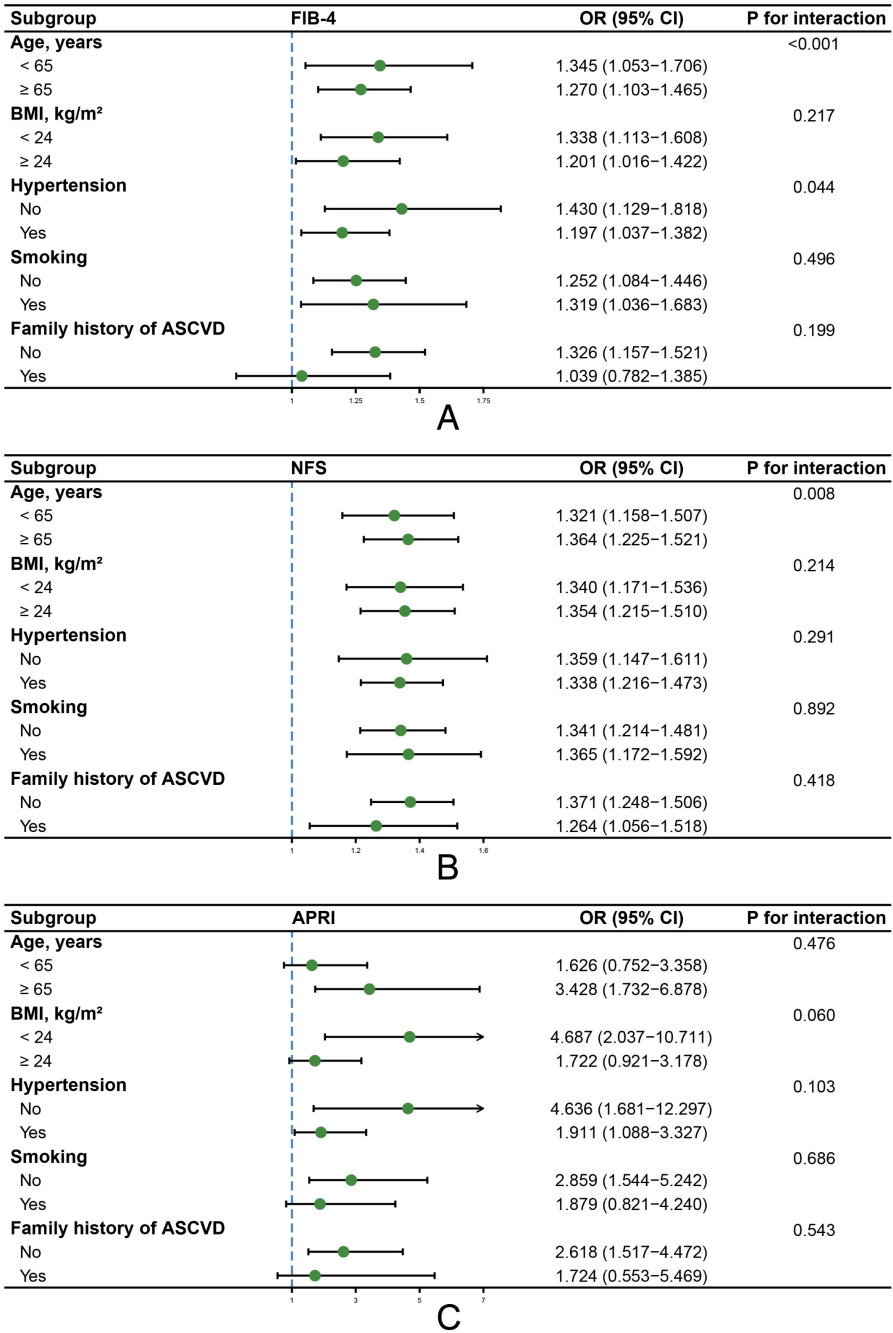
A multivariate-adjusted subgroup analysis for determining the associations between three liver fibrosis scores and the risk of having ASCVD in patients with prediabetes. **(A)** A subgroup analysis of the associations between FIB-4 and ASCVD. **(B)** A subgroup analysis of the associations between NFS and ASCVD. **(C)** A subgroup analysis of the associations between APRI and ASCVD. The analyses were adjusted for age, BMI (except for the NFS), FBG, hypertension, TC, TG, HDL-C, lipid-lowering treatment, BUN, eGFR, uric acid, smoking, regular exercise, and family history of ASCVD.

To further elucidate the relationships between liver fibrosis scores and ASCVD, we conducted a series of subgroup analyses stratified by age, BMI, hypertension status, smoking status, and family history of ASCVD (**Figure 3**). The results revealed that FIB-4 was significantly associated with ASCVD in most of the subgroups (except for the subgroup with a family history of ASCVD). Moreover, NFS exhibited stable associations with ASCVD across all of the subgroups, whereas the associations between APRI and ASCVD substantially varied across the different subgroups. In addition, interactions were detected in the age and hypertension subgroups for FIB-4, as well as in the age subgroup for NFS, whereas no evidence of interactions was detected in the remaining subgroups.

### 3.5 Diagnostic performance comparison

**Figure 4:**
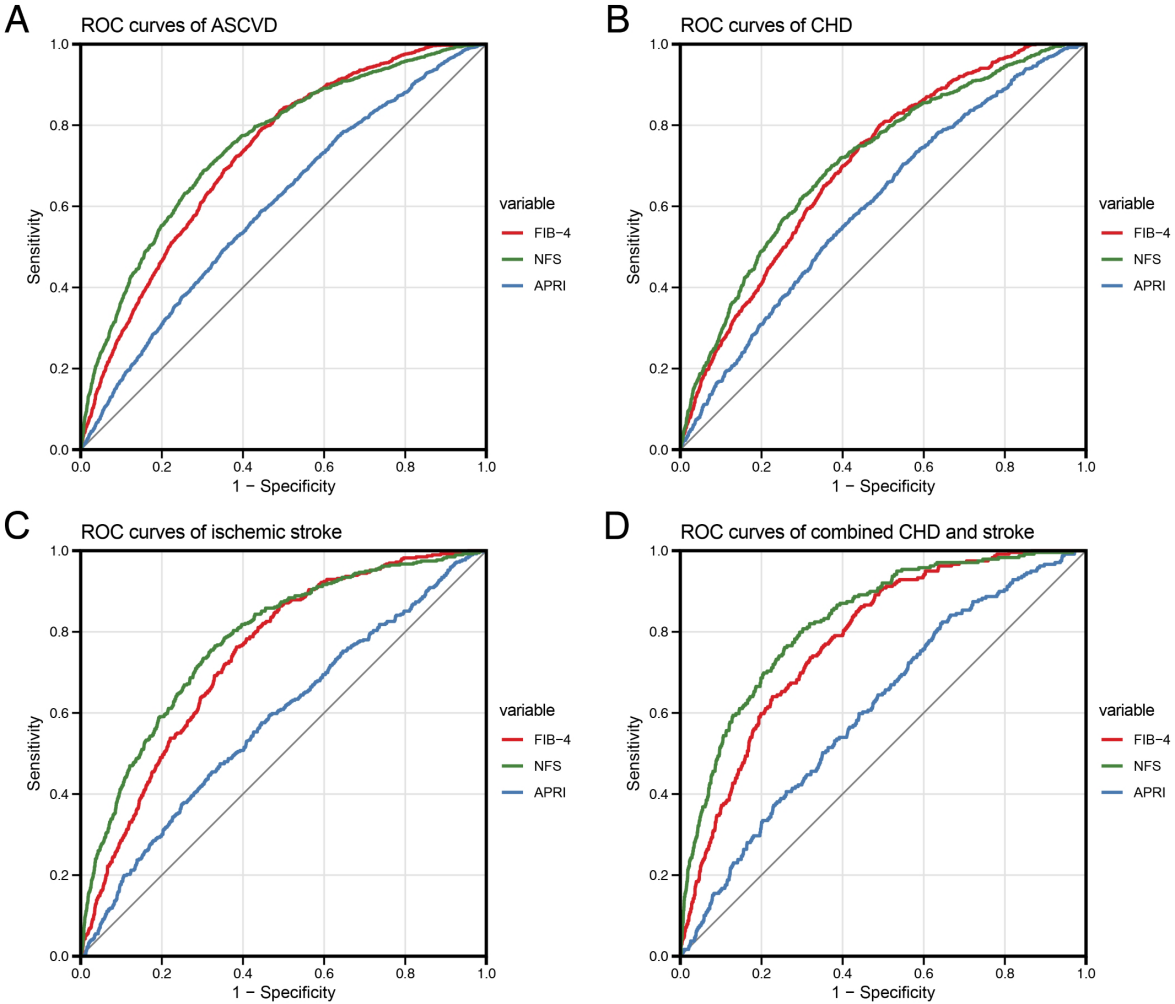
ROC curves comparing the accuracies of the three liver fibrosis scores in terms of identifying individuals with ASCVD, CHD, ischemic stroke, and combined CHD and stroke in the study population. **(A)** Performances of the liver fibrosis scores in terms of identifying individuals with ASCVD. **(B)** Performances of the liver fibrosis scores with respect to identifying individuals with CHD. **(C)** Performances of the liver fibrosis scores in terms of identifying individuals with ischemic stroke. **(D)** Performances of the liver fibrosis scores with respect to identifying individuals with combined CHD and stroke.

The ROC curve was used to assess the accuracies of the three liver fibrosis scores in terms of identifying ASCVD, CHD, ischemic stroke, and combined CHD and stroke among the patients with prediabetes in our study (**Figure 4**, **Table 3**). Both FIB-4 and NFS exhibited AUC values exceeding 0.7 in detecting individuals with ASCVD, CHD, ischemic stroke, and combined CHD and stroke. Moreover, the AUC values produced for NFS were slightly greater than those obtained for FIB-4, and the differences were observed to be statistically significant according to the results of the DeLong test (P < 0.05), with the exception of the CHD group (P = 0.109). In contrast, APRI demonstrated a significantly lower ability to detect individuals with ASCVD, CHD, ischemic stroke, and combined CHD and stroke when compared to FIB-4 and NFS, with all four AUC values being close to 0.6.

**Table 3:** ROC curve analysis for assessing the accuracies of the three liver fibrosis scores in terms of identifying ASCVD, CHD, ischemic stroke, and combined CHD and stroke.

	AUC	95% CI	P value	Sensitivity (%)	Specificity (%)	Cutoff value
ASCVD						
FIB-4	0.727	0.713–0.741	<0.001	0.791	0.554	1.207
NFS	0.751	0.737–0.765	<0.001	0.686	0.699	-0.658
APRI	0.599	0.583–0.615	<0.001	0.589	0.554	0.242
CHD						
FIB-4	0.703	0.685–0.721	<0.001	0.756	0.554	1.207
NFS	0.712	0.692–0.731	<0.001	0.688	0.639	-0.834
APRI	0.604	0.583–0.625	<0.001	0.704	0.451	0.218
Ischemic stroke						
FIB-4	0.740	0.718–0.762	<0.001	0.763	0.616	1.291
NFS	0.779	0.756–0.802	<0.001	0.765	0.669	-0.744
APRI	0.582	0.552–0.611	<0.001	0.475	0.657	0.267
Combined CHD and stroke						
FIB-4	0.780	0.754–0.805	<0.001	0.862	0.555	1.208
NFS	0.827	0.802–0.852	<0.001	0.808	0.698	-0.661
APRI	0.611	0.577–0.645	<0.001	0.820	0.359	0.200

ROC, receiver operating characteristic; ASCVD, atherosclerotic cardiovascular disease; CHD, coronary heart disease; AUC, area under the curve; CI, confidence interval; FIB-4, fibrosis-4 index; NFS, nonalcoholic fatty liver disease fibrosis score; APRI, aspartate aminotransferase-to-platelet ratio index.

The P values were derived from the comparisons between an AUC of 0.5 and the AUC values of the three liver fibrosis scores; P < 0.05 is considered to be statistically significant.

### 3.6 Sensitivity analysis

In the sensitivity analysis, after extreme liver fibrosis score values (FIB-4 and/or NFS and/or APRI values below and above the 1st and 99th percentiles, respectively) were excluded, a total of 7,080 patients were included in the study. The associations between the liver fibrosis scores and ASCVD, CHD, ischemic stroke, and combined CHD and stroke remained relatively stable after adjusting for a range of potential confounding factors (**Supplementary Table 1**). Furthermore, after patients with elevated levels of ALT and/or AST were excluded, the study sample consisted of 6,556 individuals, and the associations between the liver fibrosis scores and ASCVD, CHD, ischemic stroke, and combined CHD and stroke persisted (**Supplementary Table 2**).

## 4 Discussion

To our knowledge, this study is the first cross-sectional study to elucidate the association between liver fibrosis as assessed via liver fibrosis scores and the presence of ASCVD in patients with prediabetes, particularly in middle-aged and older individuals. Recent studies have revealed a notable association between noninvasive liver fibrosis scores as represented by the FIB-4 score and the risk of ASCVD in individuals with T2DM (9, 13). Our findings further indicated that noninvasive liver fibrosis scores (including FIB-4, NFS, and APRI values) were independently associated with an increased likelihood of having ASCVD in patients with prediabetes aged 40 to 80 years. These findings highlight the importance of liver fibrosis scores as reliable indicators for identifying the presence of ASCVD in individuals with prediabetes, thus providing guidance for clinicians to better implement risk stratification and timely intervention.

Although the pathophysiological mechanisms linking MASLD and advanced liver fibrosis to ASCVD remain to be fully elucidated, multiple pathogenic factors, such as insulin resistance, atherogenic dyslipidemia, oxidative stress, proinflammatory mediators, and a prothrombotic state, are considered to be relevant factors (16, 17). Moreover, the concept of the liver-heart axis has been proposed to elucidate the systemic interplay between the liver and heart, which is mediated by metabolic, inflammatory, and vascular pathways; additionally, key signaling molecules, including adipokines, hepatokines, and cardiomyokines, play crucial roles in facilitating this interorgan communication (16, 18). In this context, noninvasive liver fibrosis scores, such as FIB-4, effectively reflect liver fibrosis severity and are increasingly used to examine the association between MASLD-related liver fibrosis and ASCVD.

Our study did not observe any significant correlation between hepatic steatosis assessed via abdominal ultrasonography and ASCVD or its subtypes. In contrast, liver fibrosis scores, which serve as surrogate markers for liver fibrosis, were observed to be closely associated with ASCVD in our study, thus implying that in addition to conventional cardiometabolic risk factors (such as hypertension, dyslipidemia, and smoking), the adverse impact of liver fibrosis on ASCVD warrants further attention. Some studies have yielded findings consistent with ours. Khawaja et al. (19) observed no association between hepatic TG content and ASCVD in participants without T2DM, and even reported a possible protective association in those with T2DM. It was also reported that hepatic steatosis exhibited no significant association with ASCVD risk in middle-aged and older individuals as well as in patients with MASLD after adjusting for confounding variables (20). Growing evidence further suggests that liver fibrosis, as the key determinant of MASLD progression, is more strongly associated with ASCVD than other histological features, such as simple steatosis or steatohepatitis (21). In line with this, a retrospective observational study provided evidence that FIB-4 ≥ 1.3, rather than the severity of ultrasound-assessed steatosis, was an independent risk factor for all-cause mortality in diabetic patients (22). Taken together, these observations indicate that hepatic steatosis per se may not be the main determinant of ASCVD risk, whereas liver fibrosis appears more strongly associated with ASCVD.

In this study, we further assessed the correlations between different liver fibrosis scores and CHD, ischemic stroke, and combined CHD and stroke, respectively. All three liver fibrosis scores demonstrated significant values for detecting individuals with CHD, whereas only NFS could serve as a screening tool for identifying the presence of ischemic stroke or combined CHD and stroke in patients with prediabetes. The discrepancies among these results may be attributed to sample size issues, as there were only half as many patients with ischemic stroke analyzed in this study as there were patients with CHD, and the number of patients with combined CHD and stroke was even smaller. More importantly, CHD and ischemic stroke (as distinct subtypes of ASCVD) differ in their underlying pathophysiological mechanisms, which may account for their divergent associations with MASLD and hepatic fibrosis. Riley et al. (23) reported that MASLD was significantly associated with an increased risk of CHD in individuals with T2DM, whereas no such association was observed for ischemic stroke. Notably, the ability of different liver fibrosis scores to evaluate ASCVD risk may vary; moreover, based on a previous meta-analysis, NFS may offer greater discriminatory capacity for identifying individuals who are at elevated cardiovascular risk levels (24). Accordingly, it is plausible that liver fibrosis scores are differentially linked to specific ASCVD subtypes, thereby reflecting the heterogeneity of disease pathophysiology and the varying diagnostic efficacies of these indices.

According to the ROC analysis, FIB-4 and NFS were reliable discriminators of ASCVD, as both demonstrated AUC values greater than 0.7, whereas APRI was a relatively poor discriminator of ASCVD, with its AUC values being close to 0.6. Similarly, Riccio et al. (25) reported that both FIB-4 and NFS exhibited AUC values greater than 0.7 in terms of detecting individuals with nonfatal myocardial infarction. Compared with other indices, FIB-4 is generally regarded as being a superior indicator for the assessment of liver fibrosis, and the ADA clinical guidelines recommend FIB-4 as the initial test of choice for screening patients who are at high risk of advanced liver fibrosis (26, 27). However, NFS demonstrated superior performance in terms of identifying patients with ASCVD in our study population. Consistently, a prospective cohort study involving patients with biopsy-proven MASLD demonstrated that, among the same noninvasive fibrosis indicators employed in our study, NFS was the only independent predictor of cardiovascular disease (28). This result may be attributed to the inclusion of additional variables in NFS relative to FIB-4, including the BMI and albumin parameters. Indeed, obesity and hypoalbuminemia are robust predictors of ASCVD risk and the related mortality (29). Therefore, although FIB-4 remains the preferred tool for liver fibrosis screening, NFS appears to offer superior utility for detecting ASCVD. Moreover, we observed that the cutoff values of FIB-4 and APRI at the maximum Youden index were close to the thresholds for the indeterminate risk of liver fibrosis (with values of 1.3 for FIB-4 and 0.25 for APRI), which aligned with the results of the logistic regression analysis, suggesting that even indeterminate-risk liver fibrosis is significantly associated with increased ASCVD risk in middle-aged and older individuals with prediabetes.

Another interesting finding is that although the AST/ALT ratio significantly differed between the two groups, it was not independently associated with an increased risk of having ASCVD. Similarly, Li et al. (30) also reported that the AST/ALT ratio was ineffective at detecting the presence of ASCVD among patients with prediabetes. According to the ADA guidelines, a screening strategy relying on elevated aminotransferase levels would fail to identify patients with clinically significant liver fibrosis (27). Therefore, the use of composite indicators based on multiple risk factors (including liver fibrosis scores) is recommended to comprehensively assess liver fibrosis risks. The sensitivity analysis results suggested that after patients with elevated ALT and/or AST levels were excluded, the associations between the liver fibrosis scores and ASCVD remained relatively stable, thus highlighting the important notion that even individuals with normal aminotransferase levels should not underestimate the risk of liver fibrosis and the accompanying ASCVD risk.

The present study has several limitations. First, given that the health check-up data lacked information on glycated hemoglobin and 75-g oral glucose tolerance test results, only FBG was used to diagnose prediabetes. Consequently, a fraction of individuals with early diabetes may have been misclassified into our study population. Second, although the ADA guidelines recommend the use of transient elastography for further risk stratification in individuals with an FIB-4 > 1.3, the limited number of participants who underwent this assessment in our study precluded a robust analysis of the association between liver stiffness measured via transient elastography and ASCVD (27). Third, the cross-sectional design of the study precluded us from establishing a temporal or causal relationship between liver fibrosis and ASCVD in the population with prediabetes.

## 5 Conclusion

In conclusion, the findings of our study indicate that liver fibrosis assessed via noninvasive indices is strongly associated with an increased risk of having ASCVD in middle-aged and older patients with prediabetes, thus underscoring the necessity of the routine monitoring of liver fibrosis scores as a critical component of comprehensive assessments of both MASLD and the presence of ASCVD in individuals with prediabetes. Future prospective and mechanistic studies are needed to further elucidate the intricate relationship between liver fibrosis and ASCVD in individuals with prediabetes.

## Data Availability

The raw data supporting the conclusions of this article will be made available by the authors, without undue reservation.
