# Supplementary material for: Liver fibrosis assessed via noninvasive liver fibrosis scores is associated with atherosclerotic cardiovascular disease in middle-aged and older patients with prediabetes
Source: Front Endocrinol (Lausanne). 2026 Jan 9;16:1712481. doi: 10.3389/fendo.2025.1712481 (PMC12827147; doi:10.3389/fendo.2025.1712481)
Supplement: Supplementary file 1 [file Table1.docx]

Supplementary Material

# Supplementary Tables

| **Supplementary Table 1** Associations of three liver fibrosis scores with ASCVD, CHD, ischemic stroke, and combined CHD and stroke in the study population after excluding extreme liver fibrosis score values (defined as FIB-4 and/or NFS and/or APRI values below and above the 1st and 99th percentiles, respectively). | | | | | | | | |
| --- | --- | --- | --- | --- | --- | --- | --- | --- |
|  |  | |  | Univariate model | |  | Adjusted model^a^ | |
|  |  | |  | OR (95% CI) | P value |  | OR (95% CI) | P value |
| ASCVD | |  |  |  |  |  |  |  |
| FIB-4 | Continuous variable | |  | 3.321 (2.989–3.694) | <0.001 |  | 1.228 (1.052–1.433) | 0.009 |
|  | < 1.3 | |  | 1 (Reference) |  |  | 1 (Reference) |  |
|  | 1.3–2.67 | |  | 3.728 (3.270–4.256) | <0.001 |  | 1.319 (1.111–1.566) | 0.002 |
|  | ≥ 2.67 | |  | 5.973 (4.365–8.132) | <0.001 |  | 1.210 (0.825–1.766) | 0.326 |
| NFS | Continuous variable | |  | 2.558 (2.384–2.749) | <0.001 |  | 1.344 (1.227–1.473) | <0.001 |
|  | < -1.455 | |  | 1 (Reference) |  |  | 1 (Reference) |  |
|  | -1.455–0.676 | |  | 4.296 (3.600–5.159) | <0.001 |  | 1.521 (1.238–1.876) | <0.001 |
|  | ≥ 0.676 | |  | 19.055 (14.656–24.876) | <0.001 |  | 2.561 (1.860–3.530) | <0.001 |
| APRI | Continuous variable | |  | 9.352 (5.529–15.758) | <0.001 |  | 2.487 (1.288–4.769) | 0.006 |
|  | < 0.25 | |  | 1 (Reference) |  |  | 1 (Reference) |  |
|  | 0.25–0.5 | |  | 1.592 (1.407–1.801) | <0.001 |  | 1.170 (1.012–1.352) | 0.034 |
|  | ≥ 0.5 | |  | 1.748 (1.288–2.341) | <0.001 |  | 1.362 (0.947–1.935) | 0.090 |
| CHD |  | |  |  |  |  |  |  |
| FIB-4 | Continuous variable | |  | 2.794 (2.459–3.175) | <0.001 |  | 1.215 (1.010–1.459) | 0.038 |
|  | < 1.3 | |  | 1 (Reference) |  |  | 1 (Reference) |  |
|  | 1.3–2.67 | |  | 3.037 (2.572–3.593) | <0.001 |  | 1.264 (1.026–1.559) | 0.028 |
|  | ≥ 2.67 | |  | 5.217 (3.522–7.590) | <0.001 |  | 1.283 (0.812–1.998) | 0.277 |
| NFS | Continuous variable | |  | 2.149 (1.970–2.346) | <0.001 |  | 1.230 (1.100–1.375) | <0.001 |
|  | < -1.455 | |  | 1 (Reference) |  |  | 1 (Reference) |  |
|  | -1.455–0.676 | |  | 3.312 (2.680–4.129) | <0.001 |  | 1.364 (1.072–1.747) | 0.013 |
|  | ≥ 0.676 | |  | 11.366 (8.190–15.765) | <0.001 |  | 2.097 (1.423–3.086) | <0.001 |
| APRI | Continuous variable | |  | 9.768 (5.032–18.732) | <0.001 |  | 2.561 (1.171–5.520) | 0.017 |
|  | < 0.25 | |  | 1 (Reference) |  |  | 1 (Reference) |  |
|  | 0.25–0.5 | |  | 1.645 (1.401–1.932) | <0.001 |  | 1.232 (1.034–1.469) | 0.020 |
|  | ≥ 0.5 | |  | 1.804 (1.213–2.608) | 0.002 |  | 1.273 (0.820–1.927) | 0.266 |
| Ischemic stroke | |  |  |  |  |  |  |  |
| FIB-4 | Continuous variable | |  | 3.257 (2.772–3.827) | <0.001 |  | 1.187 (0.927–1.515) | 0.171 |
|  | < 1.3 | |  | 1 (Reference) |  |  | 1 (Reference) |  |
|  | 1.3–2.67 | |  | 4.638 (3.668–5.911) | <0.001 |  | 1.517 (1.133–2.040) | 0.005 |
|  | ≥ 2.67 | |  | 5.797 (3.298–9.692) | <0.001 |  | 1.008 (0.522–1.873) | 0.980 |
| NFS | Continuous variable | |  | 2.957 (2.621–3.345) | <0.001 |  | 1.480 (1.271–1.729) | <0.001 |
|  | < -1.455 | |  | 1 (Reference) |  |  | 1 (Reference) |  |
|  | -1.455–0.676 | |  | 5.337 (3.794–7.747) | <0.001 |  | 1.716 (1.175–2.569) | 0.007 |
|  | ≥ 0.676 | |  | 27.943 (18.180–43.672) | <0.001 |  | 3.200 (1.913–5.406) | <0.001 |
| APRI | Continuous variable | |  | 7.724 (3.211–18.006) | <0.001 |  | 2.348 (0.793–6.738) | 0.118 |
|  | < 0.25 | |  | 1 (Reference) |  |  | 1 (Reference) |  |
|  | 0.25–0.5 | |  | 1.506 (1.216–1.865) | <0.001 |  | 1.107 (0.870–1.408) | 0.409 |
|  | ≥ 0.5 | |  | 1.704 (0.996–2.751) | 0.039 |  | 1.504 (0.814–2.642) | 0.172 |
| Combined CHD and stroke | | |  |  |  |  |  |  |
| FIB-4 | Continuous variable | |  | 3.770 (3.088–4.602) | <0.001 |  | 1.246 (0.917–1.686) | 0.156 |
|  | < 1.3 | |  | 1 (Reference) |  |  | 1 (Reference) |  |
|  | 1.3–2.67 | |  | 5.259 (3.843–7.316) | <0.001 |  | 1.341 (0.910–1.996) | 0.143 |
|  | ≥ 2.67 | |  | 9.902 (5.327–17.528) | <0.001 |  | 1.209 (0.579–2.438) | 0.603 |
| NFS | Continuous variable | |  | 3.835 (3.262–4.535) | <0.001 |  | 1.834 (1.493–2.267) | <0.001 |
|  | < -1.455 | |  | 1 (Reference) |  |  | 1 (Reference) |  |
|  | -1.455–0.676 | |  | 11.085 (6.161–22.502) | <0.001 |  | 3.185 (1.702–6.643) | <0.001 |
|  | ≥ 0.676 | |  | 69.452 (36.012–147.779) | <0.001 |  | 6.277 (2.965–14.356) | <0.001 |
| APRI | Continuous variable | |  | 7.536 (2.406–22.240) | <0.001 |  | 1.290 (0.303–5.184) | 0.725 |
|  | < 0.25 | |  | 1 (Reference) |  |  | 1 (Reference) |  |
|  | 0.25–0.5 | |  | 1.575 (1.196–2.075) | 0.001 |  | 1.050 (0.770–1.431) | 0.756 |
|  | ≥ 0.5 | |  | 1.648 (0.796–3.051) | 0.141 |  | 1.088 (0.480–2.248) | 0.829 |
| Abbreviations: ASCVD, atherosclerotic cardiovascular disease; CHD, coronary heart disease; FIB-4, fibrosis-4 index; NFS, nonalcoholic fatty liver disease fibrosis score; APRI, aspartate aminotransferase-to-platelet ratio index; OR, odds ratio; CI, confidence interval. | | | | | | | | |
| ^a^The model was adjusted for age, BMI (except for the NFS), FBG, hypertension, TC, TG, HDL-C, lipid-lowering treatment, BUN, eGFR, uric acid, smoking, regular exercise, and family history of ASCVD. | | | | | | | | |

| **Supplementary Table 2** Associations of three liver fibrosis scores with ASCVD, CHD, ischemic stroke, and combined CHD and stroke in the study population after excluding patients with elevated ALT and/or AST levels. | | | | | | | | | |
| --- | --- | --- | --- | --- | --- | --- | --- | --- | --- |
|  |  | | |  | Univariate model | |  | Adjusted model^a^ | |
|  |  | | |  | OR (95% CI) | P value |  | OR (95% CI) | P value |
| ASCVD | |  | |  |  |  |  |  |  |
| FIB-4 | Continuous variable | | |  | 2.938 (2.664–3.244) | <0.001 |  | 1.219 (1.061–1.400) | 0.005 |
|  | < 1.3 | | |  | 1 (Reference) |  |  | 1 (Reference) |  |
|  | 1.3–2.67 | | |  | 3.715 (3.242–4.265) | <0.001 |  | 1.289 (1.077–1.543) | 0.006 |
|  | ≥ 2.67 | | |  | 7.170 (5.454–9.413) | <0.001 |  | 1.419 (1.003–2.004) | 0.047 |
| NFS | Continuous variable | | |  | 2.444 (2.283–2.618) | <0.001 |  | 1.345 (1.232–1.470) | <0.001 |
|  | < -1.455 | | |  | 1 (Reference) |  |  | 1 (Reference) |  |
|  | -1.455–0.676 | | |  | 4.574 (3.776–5.585) | <0.001 |  | 1.553 (1.243–1.951) | <0.001 |
|  | ≥ 0.676 | | |  | 19.930 (15.421–25.894) | <0.001 |  | 2.578 (1.875–3.553) | <0.001 |
| APRI | Continuous variable | | |  | 68.554 (36.229–130.018) | <0.001 |  | 3.842 (1.735–8.486) | <0.001 |
|  | < 0.25 | | |  | 1 (Reference) |  |  | 1 (Reference) |  |
|  | 0.25–0.5 | | |  | 1.879 (1.655–2.132) | <0.001 |  | 1.215 (1.045–1.410) | 0.011 |
|  | ≥ 0.5 | | |  | 3.421 (2.272–5.090) | <0.001 |  | 1.633 (0.987–2.677) | 0.053 |
| CHD | |  | |  |  |  |  |  |  |
| FIB-4 | Continuous variable | | |  | 2.462 (2.194–2.766) | <0.001 |  | 1.215 (1.030–1.431) | 0.020 |
|  | < 1.3 | | |  | 1 (Reference) |  |  | 1 (Reference) |  |
|  | 1.3–2.67 | | |  | 3.086 (2.595–3.678) | <0.001 |  | 1.281 (1.028–1.596) | 0.027 |
|  | ≥ 2.67 | | |  | 5.766 (4.068–8.080) | <0.001 |  | 1.452 (0.954–2.191) | 0.078 |
| NFS | Continuous variable | | |  | 2.035 (1.876–2.210) | <0.001 |  | 1.222 (1.098–1.360) | <0.001 |
|  | < -1.455 | | |  | 1 (Reference) |  |  | 1 (Reference) |  |
|  | -1.455–0.676 | | |  | 3.464 (2.760–4.394) | <0.001 |  | 1.378 (1.063–1.799) | 0.017 |
|  | ≥ 0.676 | | |  | 11.339 (8.256–15.612) | <0.001 |  | 2.028 (1.380–2.982) | <0.001 |
| APRI | Continuous variable | | |  | 69.039 (31.006–153.085) | <0.001 |  | 5.741 (2.236–14.608) | <0.001 |
|  | < 0.25 | | |  | 1 (Reference) |  |  | 1 (Reference) |  |
|  | 0.25–0.5 | | |  | 1.926 (1.634–2.271) | <0.001 |  | 1.306 (1.089–1.566) | 0.004 |
|  | ≥ 0.5 | | |  | 3.393 (2.004–5.521) | <0.001 |  | 1.785 (0.989–3.118) | 0.047 |
| Ischemic stroke | | |  |  |  |  |  |  |  |
| FIB-4 | Continuous variable | | |  | 2.767 (2.400–3.191) | <0.001 |  | 1.160 (0.930–1.442) | 0.184 |
|  | < 1.3 | | |  | 1 (Reference) |  |  | 1 (Reference) |  |
|  | 1.3–2.67 | | |  | 4.404 (3.454–5.659) | <0.001 |  | 1.341 (0.988–1.829) | 0.061 |
|  | ≥ 2.67 | | |  | 7.612 (4.813–11.758) | <0.001 |  | 1.265 (0.719–2.188) | 0.406 |
| NFS | Continuous variable | | |  | 2.704 (2.421–3.029) | <0.001 |  | 1.462 (1.265–1.693) | <0.001 |
|  | < -1.455 | | |  | 1 (Reference) |  |  | 1 (Reference) |  |
|  | -1.455–0.676 | | |  | 5.777 (3.965–8.760) | <0.001 |  | 1.740 (1.145–2.729) | 0.012 |
|  | ≥ 0.676 | | |  | 30.015 (19.350–47.886) | <0.001 |  | 3.241 (1.908–5.611) | <0.001 |
| APRI | Continuous variable | | |  | 39.444 (13.671–111.020) | <0.001 |  | 1.854 (0.497–6.752) | 0.353 |
|  | < 0.25 | | |  | 1 (Reference) |  |  | 1 (Reference) |  |
|  | 0.25–0.5 | | |  | 1.742 (1.400–2.166) | <0.001 |  | 1.100 (0.857–1.410) | 0.453 |
|  | ≥ 0.5 | | |  | 3.147 (1.550–5.822) | <0.001 |  | 1.657 (0.714–3.545) | 0.214 |
| Combined CHD and stroke | | | |  |  |  |  |  |  |
| FIB-4 | Continuous variable | | |  | 3.107 (2.633–3.670) | <0.001 |  | 1.258 (0.972–1.624) | 0.080 |
|  | < 1.3 | | |  | 1 (Reference) |  |  | 1 (Reference) |  |
|  | 1.3–2.67 | | |  | 5.279 (3.822–7.420) | <0.001 |  | 1.339 (0.894–2.026) | 0.161 |
|  | ≥ 2.67 | | |  | 12.782 (7.612–21.052) | <0.001 |  | 1.521 (0.791–2.884) | 0.203 |
| NFS | Continuous variable | | |  | 3.200 (2.788–3.689) | <0.001 |  | 1.683 (1.399–2.032) | <0.001 |
|  | < -1.455 | | |  | 1 (Reference) |  |  | 1 (Reference) |  |
|  | -1.455–0.676 | | |  | 14.033 (7.084–33.145) | <0.001 |  | 3.869 (1.876–9.373) | <0.001 |
|  | ≥ 0.676 | | |  | 90.045 (43.561–218.419) | <0.001 |  | 7.536 (3.315–19.538) | <0.001 |
| APRI | Continuous variable | | |  | 97.410 (27.783–328.693) | <0.001 |  | 2.639 (0.544–12.453) | 0.224 |
|  | < 0.25 | | |  | 1 (Reference) |  |  | 1 (Reference) |  |
|  | 0.25–0.5 | | |  | 1.969 (1.495–2.594) | <0.001 |  | 1.122 (0.819–1.533) | 0.473 |
|  | ≥ 0.5 | | |  | 3.988 (1.730–8.052) | <0.001 |  | 1.189 (0.438–2.907) | 0.717 |
| Abbreviations: ASCVD, atherosclerotic cardiovascular disease; CHD, coronary heart disease; ALT, alanine aminotransferase; AST, aspartate aminotransferase; OR, odds ratio; CI, confidence interval; FIB-4, fibrosis-4 index; NFS, nonalcoholic fatty liver disease fibrosis score; APRI, aspartate aminotransferase-to-platelet ratio index. | | | | | | | | | |
| ^a^The model was adjusted for age, BMI (except for the NFS), FBG, hypertension, TC, TG, HDL-C, lipid-lowering treatment, BUN, eGFR, uric acid, smoking, regular exercise, and family history of ASCVD. | | | | | | | | | |
